# Risk communication and trust in decision-maker action: a case study of the Giant Mine Remediation Plan

**DOI:** 10.3402/ijch.v72i0.21184

**Published:** 2013-08-05

**Authors:** Cynthia G. Jardine, Laura Banfield, S. Michelle Driedger, Christopher M. Furgal

**Affiliations:** 1 School of Public Health, University of Alberta, Edmonton, Alberta, Canada; 2 Health Sciences Library, McMaster University, Hamilton, Ontario, Canada; 3 Department of Community Health Sciences, Faculty of Medicine, University of Manitoba, Winnipeg, Manitoba, Canada; 4 Department of Environmental Resource Sciences/Studies, Trent University, Peterborough, Ontario, Canada; 5 Department of Indigenous Studies, Trent University, Peterborough, Ontario, Canada

**Keywords:** dual-mode model of trust and confidence, Aboriginal, mining remediation, government

## Abstract

**Background:**

The development and implementation of a remediation plan for the residual arsenic trioxide stored at the former Giant Mine site in the Canadian Northwest Territories has raised important issues related to trust. Social and individual trust of those responsible for making decisions on risks is critically important in community judgements on risk and the acceptability of risk management decisions. Trust is known to be affected by value similarity and confidence in past performance, which serve as interacting sources of cooperation in acting toward a common goal.

**Objective:**

To explore the elements of trust associated with the development and implementation of the Giant Mine Remediation Plan.

**Design:**

Semi-structured interviews were conducted with eight purposively selected key informants representing both various interested and affected parties and the two government proponents.

**Results:**

Five primary issues related to trust were identified by the participants: (1) a historical legacy of mistrust between the community (particularly Aboriginal peoples) and government; (2) barriers to building trust with the federal government; (3) limited community input and control over the decision-making process; (4) the conflicted and confounded role of the government agencies being both proponent and regulator, and the resulting need for independent oversight; and (5) distrust of the government to commit to the perpetual care required for the remediation option selected.

**Conclusions:**

The dual-mode model of trust and confidence was shown to be a useful framework for understanding the pivotal role of trust in the development of the Giant Mine Remediation Plan. Failure to recognize issues of trust based on value dissimilarity and lack of confidence based on past performance have resulted in a lack of cooperation characterized by delayed remediation and a prolonged and expensive consultation process. Government recognition of the importance of trust to these issues will hopefully improve future communication and public engagement endeavours.

Social and individual trust in decision-makers is known to play a profound role in the effectiveness of consultation around health and environmental risk issues and acceptability of decisions for risk actions (1,2). Trust in those making risk decisions is of critical importance to the development of appropriate and acceptable options for the management of those risks. Effective, reciprocal risk communication has been shown to play a major role in establishing and maintaining trust (3). Incorporation of a fair, open process of public participation and dialogue has been further advocated as an important means of increasing public trust (4).

However, the nature of this trust is very fragile. As noted by Slovic (5), trust is not automatic, nor everlasting—it is difficult to gain, even harder to maintain, and once lost almost impossible to regain. It is thus easy to understand how a postcolonial legacy of cumulative mistrust of decision-makers, such as exists amongst many Aboriginal peoples in Canada's North (6), may present enormous challenges in risk consultation and decision-making. Using a qualitative approach, the research reported here explored the elements of trust associated with the development and implementation of the Giant Mine Remediation Plan in the Canadian Northwest Territories (Fig. 1), where residual arsenic trioxide continues to pose a risk to human health and the environment.

**Fig. 1:**
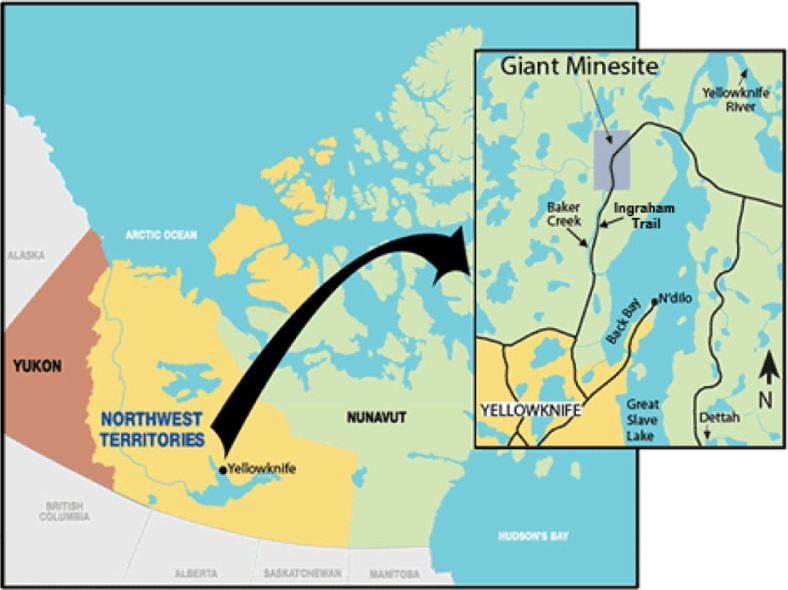
Location of Giant Mine (Aboriginal Affairs and Northern Development Canada, 2010; copy of an official work published by the Government of Canada—not produced in affiliation with, or with the endorsement of the government of Canada).

In recent years, the dual-mode model of trust and confidence (7,8), which is also known as the trust, confidence, and cooperation (TCC) model (1), has found increasing salience as a means of understanding the role of trust in risk communication and risk management. This model (outlined in Fig. 2) looks at trust (based on value similarity) and confidence (based on past performance) as interacting sources of cooperation in acting toward a common goal. This case study examination of the issues of trust and environmental health risk communication was situated within this conceptual framework.

**Fig. 2:**
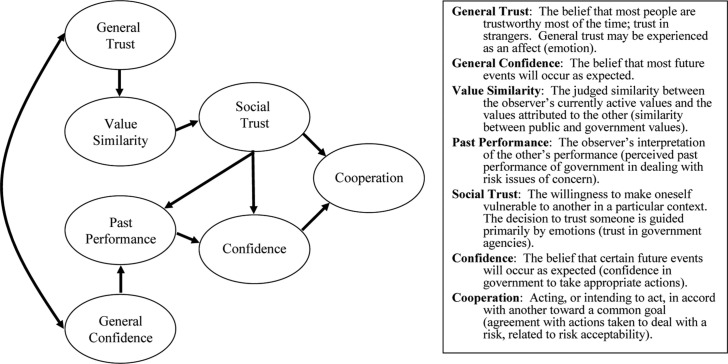
Dual-mode model of trust and confidence (after ref. 7).

## Background

This research focused on risk communication and trust surrounding the development and implementation of a plan to remediate residual arsenic trioxide contamination at a former gold mine. A history of Giant Mine and the development of the remediation plan are provided in Table 1. Various groups have been established during this process to assist with consultation and oversight (9,10). The Giant Mine Community Alliance (GMCA) was established in 2003 to share information about the project and to relay public concerns and issues about the remediation. It currently includes representatives from the city of Yellowknife, the Northwest Territories Mining Heritage Society, the Northern Territories of Federation of Labour, and the Yellowknife Chamber of Commerce, with the Yellowknives Dene First Nation (YKDFN) and the North Slave Métis Alliance (NSMA) participating as observers. The Giant Mine Advisory committee was established in 2012 to provide a forum for the Giant Mine Project Team to engage with the YKDFN. An ad hoc Oversight Working Group was also struck in 2012 to explore establishing an arm's length committee to monitor and advise on environmental aspects of the project implementation. It includes representatives from Aboriginal Affairs and Northern Development Canada (AANDC), the government of the Northwest Territories (GNWT), the city of Yellowknife, YKDFN, and Alternatives North.

**Table 1:** A history of Giant Mine and the Giant Mine Remediation Plan development^1^

Time	Events
1948–1999	Gold is mined at the Giant Mine site near Yellowknife. Giant Mine is owned by several companies during this period. In 1990, Royal Oak Resources Ltd. purchases Giant Mine, and forms Royal Oak Mines Inc. In May 1992, local workers go on strike, during which a deliberately-set explosion underground kills nine miners.
1999	Royal Oak Mines, Inc. goes into receivership. The courts transfer Giant Mine to the Government of Canada through Indian and Northern Affairs Canada (INAC). INAC strikes an agreement with Miramar Giant Mine Ltd. to provide care and maintenance at the site.
2005	Miramar terminates the agreement, and the Government of Canada enters into a cooperation agreement with the Government of the Northwest Territories (GNWT) to remediate the site.
2007	A remediation plan is developed in consultation with an 11–person, independent review panel. It calls for the long-term storage and maintenance of the 237,000 tonnes of arsenic trioxide dust using the “frozen block method,” whereby designated areas around and within each of the underground chambers and stopes will be frozen and kept frozen over the long term using thermosyphons. This plan is submitted to the Mackenzie Valley Land and Water Board (MVLWB) as part of a water licence application on October 19, 2007. The MVLWB deem the plan unlikely to be the cause of adverse environmental impact or public concern and thus not requiring an environmental assessment (EA).
2008	The City of Yellowknife (in an unprecedented partnership with the Yellowknives Dene First Nation [YKDFN] and a local environmental advocate) express concern about the potential environmental impacts of the remediation plan. In March 2008, the proposal is referred to the Mackenzie Valley Environmental Impact Review Board (MVEIRB) for EA.
2010	The Developer's Assessment Report (DAR) documenting the EA is released in October 2010. In November 2010, INAC makes $250,000 available to facilitate the participation of groups and individuals in the EA process. Funding is awarded to the YKDFN, Alternatives North, and the North Slave Metis Alliance (NSMA) in January 2011.
2012	Public hearings on the EA are held on September 10–14, 2012.

1 The information reported here and related documents may be found on the Mackenzie Valley Review Board Giant Mine Project Public Registry (9) and the AANDC website (10).

## Methods

This study is one component of a larger project examining the impact of risk communication activities on public trust across three case studies involving First Nations, Inuit, and Metis populations in Canada. The interviews conducted for this portion of the research also formed the basis of a separate project examining the effectiveness of community consultation information for the remediation plan development and implementation process.

Semi-structured interviews were conducted with purposively selected key informants from November 2010 to February 2011. Questions related to trust in the proponents and in the consultation process were posed. The key informants interviewed included representatives from those deemed to be “interested and affected parties” to this issue based on the organizations and agencies involved in the events outlined in the Background section. This included the YKDFN, City of Yellowknife, the GCMA (two representatives), the Native Women's Association of the Northwest Territories, and Alternatives North. (Note: Although the NSMA was also identified as an interested and affected party, no representative from this organization was available during the two interview periods.) Also interviewed were representatives of the two government proponents involved with the development of the remediation plant: AANDC (then INAC) and GNWT.

The interviews were digitally recorded (with permission) and transcribed verbatim. Interviews were inductively coded using a constant-comparative and concept-development approach (11) based on emergent themes that evolved through the course of the research. Interpretation validity was addressed using member checking (12), whereby all participants were provided with the opportunity to verify the full transcript of their interview and to view how their specific comments would be used in the context of the full set of themes developed from the collective results.

Ethical approval for this research was obtained from the University of Alberta Health Research Ethics Board, Panel B. In addition, a Northwest Territories (NWT) Scientific Research Licence was obtained through the Aurora Research Institute (Application #1562).

## Results

Although the key informants spoke about the Giant Mine Remediation Plan development process and implementation from different perspectives, they collectively raised several common issues related to trust, or lack thereof, of notable significance. These are described here, illustrated by comments representative of the discussions. To preserve the anonymity of the interviewees, they are identified as either a government proponent or an interested and/or affected party.

### Historical legacy of mistrust

Most interviewees (government and non-government) talked about a general population distrust of government, and how this puts government in a deficit position when trying to take action on environmental issues and consult with the public:

And we distrust government so much anyways, you know … I mean the reason why we have this situation is because there's this hopeless management regime, right? This blind eye to the mining thing, terrible historical relationship with the Aboriginal people; just awful. So these people [government] come really weighted, whether they know it or not, right?

Interested and/or affected party

Specific reference was made by several people to the historical legacy of distrust between First Nations people and federal government agencies that has been reinforced through years of colonial domination, assimilation, and not honouring treaty obligations (13). Failure to recognize this prevailing attitude, and to specifically account for it in communications and consultations, was cited as an explicit reason that people viewed the development of a remediation plan from an initial position of mistrust:

There's longstanding issues dealing with the federal government, of course, [that] no one really can deny; it doesn't really matter what the issue is, whether it be environmental or whether it be an education issue or whether it be a residential school, the payouts. I mean there's a real lack of trust. And how you build up trust with a group or with the First Nations in this region that for so long, there's been a lack of trust, is a challenge, I think, and I don't know how you do that.

Interested and/or affected party

Participants acknowledged that they might have trust in specific individuals, but still distrust the organization:

Like, I trust the people that work there to want to do the right thing, but institutionally, for me this is a broken organization. As a department called Indian and Northern Affairs, they really don't give a lot of shit about Indians, you know? It's all about Northern Affairs, right? When it comes down to a conflict between development or First Nations, you can guess who loses out every time. This organization has never done anything to protect the rights of the people that sign treaty.

Interested and/or affected party

Much of their distrust was generated from a perceived government emphasis on development over the environment and the welfare of those affected. This was recognized by both the interested and/or affected parties and the government proponents:

Historically, I think a lot of people believe that when it comes to the environment and development or the economy, the environment's always been second … And so I think we need to turn that around and work towards people saying, “Oh, the government's doing that project? Oh, good. I've got confidence in them. I can rest [easy] because I know they're representing my interests.” We need to rebuild that, because I think that's been eroded over the years.

Government proponent

Other participants noted the inherent conflict in wanting to trust the government to “look after them” and make the right decisions but not being able to trust that they will do so responsibly based on their past performance:

I think that the general population like myself know they don't know anything about this stuff, don't have the time to sit and analyze whether the frozen block method is better than this other method, and that they're trusting the people who get paid to do this stuff to do the job and do it right.

Interested and/or affected party

And there're the other people that say, “Oh, my God, it's the government. We can't trust them. Who is independently going to watch this?”

Interested and/or affected party

However, it was encouraging that the government proponents interviewed in this study recognized the critical importance of community trust in carrying out their responsibilities and the need to strive to change the inherent distrust within the community that has been historically accumulated:

Our ability to implement this project revolves around our ability to get the community to trust us. I think that's one of our biggest things that we have to work with, is to get some trust or at least some buy-in.

Government proponent

### Barriers to building trust relationships with the federal government

Several people mentioned the difficulty of trying to build trust relationships with federal government representatives who either do not reside in the community or who may suddenly be re-assigned, either in terms of responsibility for a specific project or to another location:

I think just go back to the idea of trust and that people [want to know] that this person is going to be around for a while … Because the people being consulted, namely the Aboriginal people in Yellowknife or in the north, they don't want to waste their time informing some bureaucrat who's going to get on the next plane out of town and the next time it's probably going to be someone different.

Interested and/or affected party

### Limited input into decisions

Several people commented that the decision on the remediation option was made without any consultation or input from those affected by the decision, and that the subsequent consultation process has been perfunctory only:

Look, they've already made up their mind. They're freezing that stuff come hell or high water and they don't care what anybody quite frankly says at this point. And I think that because the way that this thing has rolled out, people quite frankly just don't trust them, and if you don't have trust you're not going to get anywhere.

Interested and/or affected party

You know, where I lost trust in them in this process was where, prior to making a decision about the frozen block, there were a number of meetings and then public displays of the various methods. [It was] when we heard the decision with frozen block we started doubting the evaluations of those risk assessments, that people started calling just for an independent evaluation of them.

Interested and/or affected party

There was also concern that the opportunities for consultation had not provided people with a meaningful “voice” in the discussions, making it difficult to trust the process:

Well, for me personally, I want to go away from a process feeling even if I didn't get my way, that at least I had the opportunity to be heard and it was done fairly and okay, we can agree to disagree and let's move on and okay, some of my stuff is in there but maybe not everything I wanted. But that's not how I feel now.

Interested and/or affected party

A meeting and stuff on boards isn't necessarily consultation. It's actually visiting people over time, and a respect, a respect for those Aboriginal governments.

Interested and/or affected party

### Proponent versus regulator (need for independent oversight)

The circumstances that made INAC (now AANDC) and GNWT responsible for the remediation of the former Giant Mine site puts them in the role of being both the proponent of the remediation plan and the agencies responsible for ensuring that the remediation implementation and long-term monitoring meet government regulations. A key area of distrust expressed by the interviewees revolves around the inappropriateness of a government agency playing the roles of both proponent and regulator and the need for independent oversight of the project:

DIAND [Dept. of Indian Affairs and Northern Development, now AANDC] is wearing way too many hats on this particular job. They are the proponent, the co-proponent. The minister is going to sign off on the regulatory approvals. I want to make sure that there's some good independent oversight because I don't trust DIAND and I don't really trust GNWT to be doing the inspections.

Interested and/or affected party

There are people that would say, “How can the government watch the government?”—and they have.

Interested and/or affected party

And if this site is going to exist forever, that trust has to be there, and at this point, as far as I'm concerned, only an independent body can do.

Interested and/or affected party

This confounded role was also recognized by the government proponents:

So it's a challenging role for government because of the fact that you are both, in the eyes of the public, general governments; the regulator and the proponent as well, so that brings issues with it … But we do try because, of course, that goes straight to confidence in the process on the part of the public. So yeah, it's challenging.

Government proponent

### Perpetual care commitment?

The implementation of the frozen block method for remediation requires that the thermosyphons be maintained in perpetuity. Given the transient nature of elected governments and very visible budget cutbacks to long-term programs in recent years, people do not trust that government can be depended upon to provide this commitment to perpetual care:

So, because that's there forever, freezing it, that's not a solution. That is going to require perpetual care. Somebody is going to have to be watching and monitoring data from now until eternity to make sure that stuff doesn't come unfrozen. And if it does, what do you do about it and what's the trigger point for actually doing something about it? Is there somebody going to have the resources to do it five thousand years from now? Are they going to know what to do?

Interested and/or affected party

## Discussion

The information and opinions provided by these key informants on the Giant Mine Remediation Plan all speak directly to the elements of general trust and general confidence in the dual-mode model (Fig. 2) and demonstrate how these have led to a lack of cooperation.

### General trust

Under the dual-mode model of trust and confidence, general and social trust is related to the judged similarity in values between the interested and/or affected parties and the government proponents. The respondents in this study spoke to several tenets and beliefs related to commonly-held human, moral, and justice values that they believed had been compromised in the development of the Giant Mine Remediation Plan and, therefore, influenced the sense of trust they had in government decision makers.

First and foremost, there was a generally expressed feeling that government values resource development and the economy over the protection of the environment and the well-being of people, particularly of First Nations. This is contrary to a deeply-held value that protecting the health of Canadians should be paramount in government decision making—a belief that is espoused by Health Canada in its primary objective to “prevent and reduce risks to individual health and the overall environment by working with others in a manner that fosters the trust of Canadians” (14). Valuing both health and environment also relates directly to a traditional holistic worldview of First Nations people that everything is connected, and the health of the environment and the health of their people are thereby inextricably linked (15).

Government acting as both the proponent and regulator in the Giant Mine remediation planning and implementation violates values related to organizational justice (fairness of process) and procedural justice (control over the process). This conflicted dual government role has contributed to people questioning the integrity of the chosen remediation plan and the regulatory process governing its approval and implementation. Feelings of injustice have been reinforced by government resistance to the implementation of independent oversight.

The non-proponent respondents also felt that values related to democratic decision-making have been ignored in this process. People do not think they were adequately consulted on making the “right” decision for remediation of the Giant Mine site, and that they have subsequently not been given an opportunity to have a meaningful “voice” in assessing the potential environmental impact of the frozen block method. Lack of voice is equated to lack of empowerment and lack of control over decisions that directly affect peoples’ health and well-being, thus circumventing yet a further deeply held value in a democratic society (16).

Interestingly, people seemed able to separate their trust in individuals from their social trust of government, speaking to their valuation of personal integrity and commitment over bureaucratic actions. However, building trust relations with specific members of government has been challenged by non-continuity and regular turnover of responsible staff—a pattern that is even more problematic in the North than elsewhere in Canada. Consequently, there is a general wariness that the individuals who have earned trust with the public will continue to be involved in the project and that promises of perpetual care of the site will be honoured.

### General confidence

Government's past performance, particularly with Aboriginal people in the North, has greatly impacted the confidence people have in government agencies to make good decisions. “No Canadian acquainted with the policies of domination and assimilation wonders why Aboriginal people distrust the good intentions of non-Aboriginal people and their governments today (17).” The dissonance in values leading to a lack of social trust, much of which is linked to past performance, further contributes to a lack of confidence that government has made (or will make) appropriate decisions and that they will responsibly implement the remediation plan.

### Cooperation

The distrust and lack of confidence in the government proponents have resulted in a lack of cooperation on furthering the Giant Mine Remediation Plan. The implementation of a remedial process has been delayed by several years, meaning that the arsenic trioxide currently stored in the underground stopes continues to leach into the surrounding environment. The ensuing consultation related to the environmental assessment has been much more prolonged and expensive than would have probably occurred if confidence and trust in government was higher and if the original communication around the remediation planning had been based on a more dialogic model.

However, somewhat ironically, the resistance to cooperation resulting from a lack of trust and confidence has led to a better recognition on the part of the federal government of the importance of improved dialogue and consultation. This has been specifically acknowledged through the recent availability of participation funding and the creation of new consultation groups. The government proponents interviewed in this study admitted that they need to reassess their assumptions of their overall role in developing and implementing remediation plans; they cannot successfully undertake this responsibility without better transparency of process, development of meaningful dialogue with those affected, and without sharing of decision-making power. In the short term, their willingness to explore means for further cooperation, if coupled with accountability and consistency in actions, may allow them to move forward with the remediation plans as trust and confidence are being rebuilt.

## Conclusions

Trust has, and continues to play, a pivotal role in the development and implementation of the Giant Mine Remediation Plan. The recognition of the circumstances that have led to this lack of trust, and the resulting actions to rectify this situation, will hopefully lead to a resolution of this issue that is more acceptable to everyone involved. Perhaps even more importantly, this sometimes painful process has provided a road map for future such endeavours and has highlighted the mutual benefit to be gained from implementing a process of more transparent risk communications based on a meaningful participatory process that entails truly giving people an opportunity for their concerns and opinions to be heard and to be considered in the decision-making process. It has also emphasized the value of paying attention to the goal of building and maintaining trust in risk communication.

Based on the constructs of trust expressed by those interviewed in this study, the dual-mode model of trust and confidence appears to provide a useful framework for understanding social trust accompanying resource development and remediation. However, this was a single study involving a limited number of participants. Examining issues of trust, confidence, and cooperation in other similar circumstances and with other populations is required to fully assess the appropriateness of this model for this increasingly important issue in the circumpolar north.
